# Inflammatory Biomarkers of Extracellular Matrix Remodeling and Disease Activity in Crohn’s Disease and Ulcerative Colitis

**DOI:** 10.3390/jcm11195907

**Published:** 2022-10-07

**Authors:** Viktor Domislovic, Joachim Høg Mortensen, Majken Lindholm, Morten Asser Kaarsdal, Marko Brinar, Ana Barisic, Tina Manon-Jensen, Zeljko Krznaric

**Affiliations:** 1 Department of Gastroenterology and Hepatology, University Hospital Center Zagreb, 10000 Zagreb, Croatia; 2 Biomarkers and Research, Nordic Bioscience A/S, 2730 Herlev, Denmark; 3 School of Medicine, University of Zagreb, 10000 Zagreb, Croatia

**Keywords:** extracellular matrix, biomarkers, collagen, inflammatory bowel disease, Crohn’s disease, ulcerative colitis

## Abstract

Extracellular matrix (ECM) homeostasis is highly affected in active inflammatory bowel disease (IBD). The aim of the study was to investigate serological biomarkers of type III, IV, and V collagen degradation and formation, and their association with disease activity in IBD. ECM remodeling serum biomarkers were measured in 162 IBD patients, 110 with Crohn’s disease (CD) and 52 with ulcerative colitis (UC), and in 29 healthy donors. Biomarkers of type III collagen degradation (C3M) and formation (PRO-C3), type IV collagen degradation (C4M) and formation (PRO-C4), and type V collagen formation (PRO-C5) were measured using ELISA. Inflammatory activity was assessed using endoscopic, clinical, and biochemical activity indices. The highest diagnostic value was identified in discriminating endoscopically moderate to severe disease in CD (PRO-C3, C3M/PRO-C3, and C4M with AUC of 0.70, 0.73, and 0.69, respectively) and UC (C3M, C3M/PRO-C3, and C4M with AUC of 0.86, 0.80, and 0.76, respectively). C4M and C3M/PRO-C3 in combination yielded AUC of 0.93 (0.66–0.90) in CD and 0.94 (0.65–0.99) in UC. This study confirmed that ECM remodeling reflected disease activity in CD and UC. A combination of C4M, C3M, and PRO-C3 biomarkers may potentially be considered as a biomarker differentiating moderate to severe endoscopic disease.

## 1. Introduction

Ulcerative colitis (UC) and Crohn’s disease (CD) are chronic inflammatory bowel diseases (IBDs) characterized by episodes of relapse and remission requiring continuous evaluation of disease activity [1,2]. Colonoscopy is the gold standard for diagnosis and disease activity monitoring but has disadvantages since it is time-consuming, invasive, and unpleasant for the patient. Serum biomarkers, as a simple and noninvasive method, may be used to improve disease activity monitoring, which can lead to early therapy adjustment or relapse prediction [3]. The currently used serum biomarkers of inflammation, such as C-reactive protein (CRP), erythrocyte sedimentation rate (ESR), and platelet count, are nonspecific and often do not correlate with endoscopic or clinical findings [3,4]. By comparison, fecal calprotectin (FC) may be useful in diagnosis, relapse prediction, and disease activity prediction [5,6,7,8]; however, FC is not always a reliable and specific biomarker of disease activity assessment [9,10,11] since it is subject to various factors such as bowel infection, nonsteroidal anti-inflammatory drugs, methods of assessment and storage, subject age, and daily variability [12,13,14].

The extracellular matrix (ECM) consists of the basement membrane (BM) and the interstitial matrix (IM) [15]. The BM is placed underneath the epithelium and endothelium, with type IV collagen being the most abundant collagen [16]. By comparison, the IM consists of various types of collagens such as types I and III [17]. ECM remodeling is the key process of tissue homeostasis in which old and dysfunctional proteins are degraded and replaced with newly synthesized proteins. Under pathological conditions, such as inflammation or fibrosis, ECM is highly affected [18,19,20,21,22,23,24,25,26,27], with matrix metalloproteinases (MMPs) as the major contributor to the intestinal tissue remodeling, which results in the production of protease-derived protein fragments or neo-epitopes [26,27,28]. Biochemical markers based on neo-epitopes in serum are receiving increased attention due to their diagnostic and prognostic potential [29,30,31]. Intestinal inflammation in IBD results in altered ECM remodeling in which the original ECM proteins are replaced with proteins of different composition [15,16,17,18,19]. This process results in a structurally and qualitatively different intestinal ECM, which alters the function of the affected organ (e.g., small or large bowel). High levels of protein degradation and formation fragments of the ECM are released into the bloodstream, where they can be measured and serve as a molecular and biochemical marker of various pathologies including inflammation in CD and UC, and may even reflect mucosal tissue integrity; therefore, such markers are potential candidates that can be used for monitoring mucosal healing as they are derived directly from the affected tissue [20,21,22,23,26,30].

The main pathophysiological mechanism in producing biomarkers of collagen degradation is through inflammation in which inflammatory cells produce proteases which degrade collagen. Biomarkers reflecting collagen degradation such as C3M and C4M are derived from matrix metalloproteinases (MMPs), which have been demonstrated to be highly involved in the mucosal damage and inflammation seen in IBD [25,30,32,33]. By comparison, collagen formation biomarkers such as PRO-C3, PRO-C4, and PRO-C5 reflect more the healing and fibrogenesis, and are produced during the process of collagen synthesis such as posttranslational modifications [25,30,34,35,36]. According to the available studies on the influence of MMPs on intestinal collagens and associations of ECM biomarkers in IBD, we decided to analyze the five following biomarkers of ECM remodeling [17,18,19,20,21,22,23,24,25,26]. The C3M biomarker assay quantifies a neo-epitope derived from MMP-9-mediated degradation of type III collagen [32]. The C4M biomarker assay quantifies a neo-epitope derived from MMP-2,9,12-mediated degradation of the type IV collagen alpha-1 chain [33]. PRO-C3 is the released N-terminal propeptide of type III collagen and quantifies type III collagen formation [34], and the PRO-C4 biomarker assay quantifies an epitope in the 7S domain of type IV collagen [35]. Finally, PRO-C5 is the C-terminal pro-peptide of type V collagen and is a biomarker of type V collagen [36]. Protein turnover was calculated as the ratio of degradation and formation, i.e., type III collagen turnover (C3M/PRO-C3) and type IV collagen turnover (C4M/PRO-C4).

There is medical need for improved noninvasive biomarkers for the assessment of IBD activity. Serological biomarkers that reflect tissue remodeling may be associated with disease activity in IBD. Therefore, the aim of the study was to investigate the serological biomarkers of collagen degradation and formation of the ECM (i.e., BM and IM) and their association with disease activity in IBD.

## 2. Materials and Methods

### 2.1. Patient Data

In this cross-sectional study, we measured five biomarkers of ECM remodeling in 162 IBD patients (110 CD and 52 UC) and 29 age- and gender-matched healthy donors (HDs). Demographic data, disease history, and therapy were obtained from electronic medical records and questionnaires. Anthropometric parameters were measured at patient inclusion in the study. Inclusion criteria were adult patients with an established diagnosis of IBD, while exclusion criteria were systemic infection, bowel infections, or inflammatory condition of other etiologies, as well as extraintestinal inflammation. CD and UC were classified according to the Montreal classification, which was obtained on patient inclusion. In CD, disease behavior was defined as B1—non-stricturing, non-penetrating; B2—structuring; and B3—penetrating; and disease extension as L1—ileum; L2—colon; L3—ileocolon; and L4—upper GI. In UC, disease extension was defined as E1—proctitis; E2—left-sided colitis; and E3—pancolitis. The study was approved by the Zagreb University Hospital Center Ethics Committee. Signed informed consent was obtained prior to study enrolment. All methods were performed in accordance with the relevant guidelines and regulations.

### 2.2. Disease Activity Definition

Disease activity was assessed at the time of blood sample analysis using endoscopic, clinical, and biochemical markers of disease activity. Simple Endoscopic Score in Crohn’s disease (SES-CD) was available for 60 CD, and modified Mayo Endoscopic Score (mMES) for 33 UC patients. The mMES score was used to add information on disease extension to the severity of inflammation [37]. On mMES calculation, the colon is divided into five segments and each segment is scored using Mayo Endoscopic Score (MES). mMES is then calculated taking maximal extension of the disease in consideration. Inflammatory activity was also defined as a combination of clinical and biochemical disease activity using Crohn’s Disease Activity Index (CDAI), partial Mayo score (pMayo), CRP, and FC. Patient stratification based on endoscopic scores was performed as follows: SES-CD (remission 0–2, mild 3–6, moderate 7–15, severe > 15), mMES (remission 0–2, mild 3–6, moderate 7–15, severe > 15), while clinical and biochemical activity was defined as CDAI ≥ 150 or CRP > 5 mg/L for CD, and pMayo > 1 or CRP > 5 mg/L for UC.

### 2.3. Biomarker Assay

Blood samples for biomarker analysis were obtained in the morning after overnight fasting. Biomarkers of type III collagen degradation (C3M) and formation (PRO-C3), type IV collagen degradation (C4M) and formation (PRO-C4), and type V collagen formation (PRO-C5) were measured in serum by competitive enzyme-linked immunosorbent assays (ELISAs) (Table 1) [32,33,34,35,36]. Briefly, 96-well plates pre-coated with streptavidin (Roche Diagnostics cat.no.11940279, Hvidovre, Denmark) were coated with a biotinylated antigen for 30 min at room temperature. All samples were diluted in incubation buffer containing 1% bovine serum albumin (cat.no. a-7906, ≥98 purity, Sigma Aldrich). Samples and controls were incubated with horseradish peroxidase-conjugated monoclonal antibodies for 1–3 h at 4 °C/20 °C or for 20 h at 4 °C with agitation at 300 rpm, according to the manufacturer’s protocols. Subsequently, tetramethylbenzidine (TMB, Kem-En-Tec cat.No.438OH, Taastrup, Denmark) was added (100 µL/well), and plates were incubated for 15 min at room temperature and agitated at 300 rpm. Stopping buffer (1% H_2_SO_4_) was added to stop the TMB reaction. After each incubation step, wells were washed with washing buffer (25 mM TRIZMA, 50 mM NaCl, 0.036% Bronidox L5, 0.1% Tween 20) using a standardized ELISA plate washing machine (BioTek^®^ Instruments, microplate washer, ELx405 Select CW, Winooski, VT, USA). An ELISA reader (VersaMAX, Molecular Devices, Wokingham Berkshire, UK) was used to read optical densities at 450 and 650 nm. A standard curve was plotted using a 4-parameter mathematical fit model. As buffers and incubation times differed between the ELISAs, the interested reader can find a detailed description of the assay methodology in the respective references [32,33,34,35,36].

### 2.4. Statistical Analysis

Categorical variables are expressed as percentages, and continuous variables as means with standard deviation or medians with interquartile range (25th and 75th percentiles), depending on the distribution. Differences between continuous variables were tested using the two-way T-test or analysis of variance (ANOVA), followed by Tukey post-hoc analysis for parametric analysis, and the Mann–Whitney U-test or Kruskal–Wallis test with post-hoc Mann–Whitney U-test for non-parametric analysis. The false discovery rate method (FDR 5%) was used for multiple comparison correction. Discriminative power of biomarkers among disease activity groups was assessed using the receiver operating characteristic curve (ROC curve) with the DeLong methodology and stepwise logistic regression analyses to calculate the diagnostic value of combined biomarkers. First, the ROC curve analysis was performed on single biomarkers to identify most relevant biomarkers. Diagnostic accuracy was calculated by the following equation: Diagnostic accuracy = ([True negatives + true positives]/[true negatives + true positives + false negatives + false positives]). Statistical analyses were performed using MedCalc. Moreover, we used principal component analysis (PCA) on scaled and centered data to investigate the association of measured biomarkers with the endoscopic activity of the disease. The results are reported as a scree plot, graph of variables, and graph of individuals in Supplementary Materials (Figure S5). The analysis was conducted in R (version 3.6.0., R Development Core Team, Vienna, Austria) using the factoextra package. Statistical tests were two-tailed, and the level of statistical significance was set at 0.05.

## 3. Results

### 3.1. Demographic Characteristics

The average age of CD and UC patients was 36 (28–46) and 37 (24–49) years, respectively, which was similar to that of HD [39.5 (33–47)]. Comparing CD to UC, there were more male patients (60% vs. 53.8%), more smokers (21.8% vs. 13.5%), and more patients with prior surgery (50% vs. 9.6%) in the CD group. In the UC group, there was a higher proportion of patients with mild disease on endoscopy. Both CD and UC groups had a similar proportion of patients with endoscopically moderate to severe disease (30% vs. 30.3%) (Table 2).

### 3.2. Collagen Biomarkers as Surrogate Markers for Disease Activity

#### 3.2.1. Endoscopic Disease Activity

Stratifying patients according to endoscopic severity (remission, mild, moderate, and severe) demonstrated that CD patients with mild or moderate and severe disease had higher C4M levels compared to patients in remission (*p* < 0.010) and HD (*p* < 0.001) (see Supplementary Figure S1). PRO-C4 serum levels were elevated in mild and moderate to severe CD patients compared to HD (*p* = 0.008) and patients in remission (*p* = 0.049). PRO-C5 was also elevated in CD patients with mild and moderate/severe disease compared to HD (*p* = 0.031). Type III collagen turnover (C3M/PRO-C3) showed higher levels in moderate and severe active disease compared to CD in remission (*p* = 0.006). Furthermore, PRO-C3 levels were lower in CD patients with moderate to severe SES-CD score compared to CD patients in endoscopic remission (*p* = 0.040), and patients in remission and with mild disease based on SES-CD had higher PRO-C3 levels compared to HD (*p* < 0.001) (see Supplementary Figure S1).

Ulcerative colitis patients with moderate to severe disease had higher C3M levels compared to mild disease or remission patients and HD. PRO-C3 levels were lower in endoscopically moderate to severe UC compared to remission and mild group, but were higher compared to HD. UC patients with moderate to severe disease activity demonstrated higher levels of type III collagen turnover biomarker (C3M/PROC3) (1.1 ± 0.4 vs. 1.97 ± 1, *p* = 0.049). C4M levels were elevated in the moderate to severe active group compared to mild and remission patients, and the HD group (*p* < 0.01). The same was true for PRO-C5. PRO-C4 demonstrated higher levels in mild and moderate to severe disease compared to HD (*p* < 0.01) (see Supplement Figure S2).

#### 3.2.2. Clinical and Biochemical Disease Activity

Regarding clinical and biochemical disease activity in CD patients, elevated C3M, C4M, PRO-C4, and C3M/PRO-C3 levels were found in active disease. PRO-C3 was elevated in the remission group compared to the active group (see Supplement Figure S3). In UC, C3M, C4M, PRO-C4, and C3M/PRO-C3 showed higher levels in the active compared to the remission and HD groups. PRO-C3 levels in UC were elevated in the active and remission groups compared to HD, and PRO-C5 in the active group compared to HD (see Supplement Figure S4).

#### 3.2.3. Diagnostic Power of Collagen Biomarkers to Discriminate between IBD Patients in Endoscopic Remission and Active Disease

**Figure 1:**
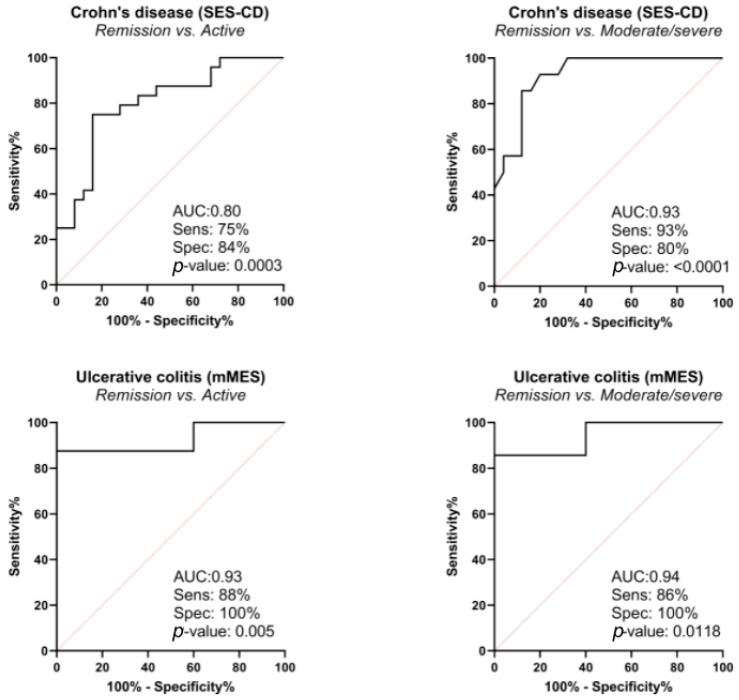
Diagnostic power of C4M, C3M/PRO-C3 biomarkers in combination by receiver operating characteristic curve (ROC curve), based on logistic regression model; correction for the following confounding factors was made: in Crohn’s disease for age, gender, body mass index (BMI), smoking, prior surgery, biological therapy, Montreal classification, and in ulcerative colitis for age, gender, BMI, biological therapy, Montreal classification.

In CD, PRO-C3, C3M/PRO-C3, and C4M had significant diagnostic value in discriminating moderate to severe disease from remission (AUC 0.70–0.73) (Table 3). These biomarkers were compared in multivariate analysis, and the best combination of biomarkers for discriminating moderate to severe disease from remission and active disease from remission was C4M and C3M/PRO-C3 (adjusted AUC 0.93 95% CI 0.66–0.90 and 0.80 95% CI 0.66–0.90, respectively) (Table 4, Figure 1).

In UC, C3M, C3M/PRO-C3, and C4M had significant diagnostic value in discriminating moderate to severe disease from remission (AUC 0.76–0.86) (Table 3). These biomarkers were compared in multivariate analysis, and the best combination of biomarkers in discriminating active disease from remission was C3M and C4M (adjusted AUC 0.95 95% CI 0.79–1), and C4M and C3M/PRO-C3 (AUC adjusted 0.93 95% CI 0.72–0.99) (Table 4, Figure 1). The combination of C4M and C3M/PROC3 yielded AUC of 0.94 95% CI 0.65–0.99.

#### 3.2.4. Principal Component Analysis of Collagen Biomarkers

PCA was performed on two sets of data: one containing patients with endoscopic remission and active disease; and one containing only patients with endoscopic remission or moderate to severe disease. In both cases, the first and second principal components explained more than 80% of the variance in the data and PRO-C3 contributed dominantly to the second principal component, while other variables (C3M, C4M, PRO-C4, and PRO-C5) contributed mainly to the first principal component. Based on the graph of individuals, it seems that patients are roughly clustered into two groups (active disease and remission) on the PC2 axis (see Supplementary Figure S5).

## 4. Discussion

This study demonstrated that serological biomarkers of collagen degradation and formation may have potential in discriminating patients with different disease activity. This was shown by performing comprehensive analysis of five ECM remodeling biomarkers (C3M, C4M, PRO-C3, PRO-C4, and PRO-C5 with calculated turnover ratios of C3M/PRO-C3 and C4M/PRO-C4) and comparison to disease activity indices at various levels; endoscopic, clinical, and biochemical, but considering endoscopic activity as the most relevant since it is the current gold standard in disease activity assessment [1,2]. This comprehensive approach was used to provide detailed analysis of both the combination and single biomarker analysis of one of the most common intestinal wall layer collagens and disease activity, which has not been undertaken so far. In addition, our study included disease extension in evaluating endoscopic disease activity. SES-CD takes disease extension into account, which is not the case for MES. Therefore, using mMES in UC is one of the important study strengths. In a study from Lobatón et al., the mMES correlated significantly with the PMS (r = 0.535), CRP, (r = 0.238), FC (r = 0.730), and histologic Geboes’ score (r = 0.615) (*p* < 0.001) [37]. This is especially important when analyzing ECM biomarkers since both disease extension and degree of inflammation have an influence on total biomarker levels, i.e., using MES would not be appropriate since it would provide information only on the intensity of inflammation, and without disease extent, interpretation of results would be questionable.

Our results demonstrated that the combination of type IV collagen degradation (C4M) and type III collagen turnover (C3M/PRO-C3 ratio) was able to discriminate patients with endoscopic remission from patients with endoscopically active or moderate to severe disease with high accuracy. First, univariate analysis on a single biomarker (Table 3) was performed to identify the most relevant biomarkers to include in multivariate analysis and to obtain the combination of biomarkers with the best discriminative power (Table 4, Figure 1). Therefore, the best combination of biomarkers was C4M and C3M/PRO-C3, with adjusted AUC of 0.93, in discriminating moderate to severe disease from remission in CD, and adjusted AUC of 0.94 in discriminating moderate to severe disease in UC. This finding is important since it highlights several facts. First, as mentioned before, every observed biomarker represents certain collagens with different roles in tissue homeostasis, and, by using a combination of biomarkers, we are in fact observing different levels of pathophysiological mechanisms in the process of inflammation at the same time [32,33,34,35,36]. Second, the ECM is a complex system whose role cannot be revealed using a single biomarker only; hence, the combination of different biomarkers is an important advantage. Finally, this is the first study to identify this specific combination of biomarkers (C4M and C3M/PRO-C3) as being potentially relevant, which should encourage future prospective studies using a larger sample.

Single biomarker analysis is also important since it indicates one pathophysiological mechanism, which, if proven relevant, may be combined in future with other biomarkers that have currently not been analyzed. Namely, as shown in Supplementary Figures S1 and S2, CD patients with endoscopically moderate to severe and mild disease had higher concentrations of C4M and PRO-C4, whereas in UC, the levels of C4M increased with inflammatory activity, and PRO-C4 had a trend towards elevation with increased disease activity. C4M is a degradation product, and the PRO-C4 formation product of type IV collagen is the most abundant collagen of BM [16]. We hypothesize that serological biomarkers of BM remodeling (e.g., C4M and PRO-C4) may serve as markers of epithelial and mucosal damage, i.e., superficial inflammation. To support this hypothesis, in UC, where superficial inflammation is present, C4M (i.e., type IV collagen degradation) was significantly higher in moderate to severe disease as compared with mild disease in the same group, which was not the case in CD. Therefore, assessing tissue damage and remodeling with this biomarker may have an advantage in disease activity monitoring and serve as a surrogate marker of mucosal tissue destruction/inflammation.

PRO-C3 (i.e., type III collagen formation) was the only biomarker that demonstrated an elevated trend in remission compared to active disease, indicating increased formation and tissue deposition of type III collagen as a healing response. Since type III collagen demonstrated increased formation (i.e., increased PRO-C3) in remission both in CD and UC, and due to its role in wound healing and fibrosis, we could hypothesize that lower PRO-C3 levels, which were obtained in our cohort, may be considered as a marker of the healing phase in IBD. A study on a liver fibrosis model indicates higher values of PRO-C3 (over 22.4 ng/mL) as a strong predictor of liver fibrosis progression, which is also a response to healing [38]. More importantly, a recent study by Lindholm et al., which was performed on 12 dextran sulfate sodium (DSS)-induced colitis rats and nine controls, supports our findings [39]. Specifically, C3M, C4M, and PRO-C4 levels increased in the DSS induction phase and declined in the healing phase without DSS, while rodent-PRO-C3 showed a declining tendency after induction of colitis and an increasing tendency after receiving regular water, resulting in increased turnover of type III collagen (C3M/PRO-C3 ratio) [39]. Overall, this points to the fact that there is predominantly increased degradation of type III collagen in active disease and increased formation in the healing phase.

Along with the existing data, our study confirmed for the first time that endoscopically active disease in UC and CD led to increased type III collagen turnover (C3M/PRO-C3), despite both C3M and PRO-C3 being elevated. In contrast, type IV collagen turnover (C4M/PRO-C4) remained the same in the active group and in remission, since both C4M and PRO-C4 tended to be similarly elevated between different activity groups.

PRO-C5, which is produced in wound repair and fibrogenesis, was elevated in both moderate and severe endoscopically active UC and CD. According to previous studies, elevated PRO-C5 levels were observed in CD patients with ileal disease, but the disease activity was defined biochemically as CRP above 5 mg/L [21]. The second study was conducted on subjects with CD and UC. Patients with CD and with clinically mild inflammatory activity had elevated levels of PRO-C5, while there was no difference in UC patients [23]. Our results are in line with previous studies, demonstrating that PRO-C5 is associated with disease activity in CD patients, and these data are also in line with the fact that type V collagen can serve as a marker of separate pathophysiological mechanism [23,31,36]. By comparison, using endoscopic indices of disease activity, elevated PRO-C5 levels were observed in UC also, which has not been observed before using clinical indices of disease activity only.

In addition, comparing endoscopy (Supplementary Figures S1 and S2) with clinical and biochemical disease activity (Supplementary Figures S3 and S4), we can conclude that, in a practical sense, by using only clinical and biochemical indicators of inflammatory activity some processes of ECM remodeling might be overlooked and remain unrecognized, such as PRO-C5 in UC. This is an expected and well-known disadvantage of clinical and biochemical indices, which are not specific (but are significantly easier to obtain). Therefore, in future studies, it would be desirable to use endoscopy with a clearly defined method of determining inflammatory activity.

Finally, the potential clinical relevance of ECM biomarkers was shown in a recent pilot study on CD patients, which aimed to compare serum levels of collagen formation and degradation markers between responders and non-responders to infliximab (*n* = 21) and adalimumab (*n* = 21) induction therapy [40]. This study is the first to show that the clinical response to anti-tumor necrosis factor therapy can be predicted by measuring C4M at baseline and C3M during induction; however, further studies on a larger sample are needed [40].

There were several limitations to our study. First, our IBD cohort came from a tertiary center with experienced and more complicated IBD patients (more than half of them treated with biologics, and 50% of CD patients experienced surgery). Therefore, these data should not be extrapolated to the general IBD population. However, previous surgery was considered in regression analysis to minimize this potential confounding effect. Second, the fistulizing phenotype can affect ECM biomarker levels; therefore, adjustment for disease phenotype was also made in multivariate analysis [21,22]. Next, endoscopy was available in a subgroup of patients, which could be considered a limitation. However, since the data on endoscopic disease activity in ECM studies are limited and the endoscopy is difficult to obtain, we would like to emphasize the use of endoscopic indices, which are the gold standard in evaluating disease activity, as one of the study strengths, especially using disease extension, which has not been performed before. Finally, histopathological scores and histomorphology were not obtained, which would be useful to provide additional insights into the ECM pathophysiology and is another potential contribution that may be considered in future studies.

## 5. Conclusions

In conclusion, this study confirmed that ECM remodeling quantified by serological biomarkers of collagen degradation and formation, i.e., extracellular matrix remodeling, reflects disease activity in both UC and CD. This is especially evident for collagen type III turnover, type IV collagen degradation and formation, and type V collagen. A combination of the C4M, C3M, and PRO-C3 biomarkers was demonstrated to have superior diagnostic accuracy in differentiating CD and UC patients in endoscopic remission from CD and UC patients with endoscopically active disease. Therefore, we presented ECM biomarkers as potentially significant biomarkers to monitor disease activity at a different pathophysiological level, focusing on collagen homeostasis. Future research should be undertaken using a prospective study on a larger sample of patients, and with additional ECM biomarkers to provide even more detailed insights into role of ECM in inflammation in IBD.

## Figures and Tables

**Table 1:** Overview of the ECM neo-epitope biomarkers.

Biomarker	Neo-Epitope Antigen	Description	Reference
C3M	KNGETGPQGP	MMP-mediated degradation of type III collagen	[32]
C4M	ILGHVPGMLLKGER	MMP-mediated degradation of type IV collagen	[33]
PRO-C3	CPTGPQNYSP.	Formation of new type III collagen	[34]
PRO-C4	KNGETGPQGP	Remodeling of type IV collagen	[35]
PRO-C5	PGEILGHVPG.	Formation of new type V collagen	[36]

Periods (.) depicts the neo-epitope antigen and where the antibody binds.

**Table 2:** Demographic characteristics of patient populations and healthy donors.

Variable	CD (*n* = 110)	UC (*n* = 52)	HD (*n* = 29)
Age, years (IQR)	36 (28–46)	37 (24–49)	39.5 (33–47)
Male gender, *n* (%)	66 (60.0)	28 (53.8)	17 (58.6)
BMI, kg/m^2^ (IQR)	22.57 (20–27)	23.86 (21–28)	
Smoking, *n* (%)	24 (21.8)	7 (13.5)	
Localization CD, *n* (%) L1/L2/L3/L4	20 (18.2)/17 (15.5)/73 (66.4)/6 (5.5)	-	
Behavior CD, *n* (%) B1/B2/B3	39(35.5)/42 (38.2)/29(26.3)	-	
Extension UC, *n* (%) E1/E2/E3	-	4 (7.7)/12 (23.1)/36 (69.2)	
Perianal disease, *n* (%)	37 (33.6)	-	
Endoscopic activity, *n* (%) Remission Mild Moderate to severe	32 (53.3) 10 (16.7) 18 (30.0)	10 (30.3) 13 (39.4) 10 (30.3)	
Clinical and biochemical activity, *n* (%) Remission Active	51 (46.4) 59 (53.6)	22 (42.3) 30 (57.7)	
Prior surgery, *n* (%)	55 (50.0)	5 (9.6)	
Immunosuppressive therapy, *n* (%)	33 (30.0)	16 (30.8)	
Biological therapy, *n* (%)	65 (59.1)	28 (53.8)	
Steroid therapy, *n* (%)	7 (6.3)	10 (19.2)	
CRP, mg/L	2.5 (0.8–5.7)	3.2 (0.7–8.6)	

CD, Crohn’s disease; UC, ulcerative colitis; HD, healthy donors; HDhdHDIQR, interquartile range; BMI, body mass index; CRP, C-reactive protein; L1—ileum, L2—colon, L3—ileocolon, L4—upper GI, B1—non-stricturing, non-penetrating, B2—stricturing, B3—penetrating, E1—proctitis, E2—left-sided colitis, E3—pancolitis; endoscopic activity—SES-CD for CD (remission = 0–2, mild = 3–6, moderate 7–15, severe > 15); mMES for UC (remission 0–2, mild 3–6, moderate 7–15, severe > 15); clinical and biochemical activity—CDAI ≥ 150 or CRP > 5 mg/L for CD and pMayo > 1 or CRP > 5 mg/L for UC.

**Table 3:** Diagnostic power of single collagen biomarkers to discriminate between inflammatory bowel disease patients in endoscopic remission and active disease.

Biomarker	Crohn’s Disease		Ulcerative Colitis	
Remission vs. Active	AUC (95% CI) (Sens;Spec)	*p*	AUC (95% CI) (Sens;Spec)	*p*
C3M	0.56 (0.41–0.69) (45;73)	0.477	0.64 (0.40–0.83) (44;80)	0.303
PRO-C3	0.63 (0.48–0.76) (96;31)	0.096	0.65 (0.41–0.84) (94;40)	0.337
C3M/PRO-C3	0.64 (0.50–0.77) (54;73)	0.071	0.63 (0.40–0.83) (63;80)	0.309
C4M	0.69 (0.56–0.80) (86;44)	0.006 *	0.63 (0.44–0.79) (52;80)	0.218
PRO-C4	0.61 (0.48–0.73) (46;84)	0.134	0.60 (0.41–0.76) (52;80)	0.327
PRO-C5	0.58 (0.44–0.70) (39;84)	0.310	0.56 (0.37–0.73) (18;90)	0.593
Remission vs. moderate and severe				
C3M	0.63 (0.46–0.78) (64;73)	0.184	0.86 (0.54–0.98) (71;60)	0.002 *
PRO-C3	0.70 (0.53–0.83) (79;38)	0.028 *	0.68 (0.36–0.91) (43;80)	0.263
C3M/PRO-C3	0.73 (0.56–0.85) (64;73)	0.007 *	0.80 (0.48–0.97) (86;80)	0.037 *
C4M	0.69 (0.54–0.81) (50;81)	0.018 *	0.76 (0.48–0.96) (70;80)	0.038 *
PRO-C4	0.58 (0.43–0.71) (44;84)	0.392	0.66 (0.41–0.85) (60;90)	0.239
PRO-C5	0.53 (0.38–0.67) (33;91)	0.704	0.73 (0.48–0.90) (67;80)	0.073

CRP, C-reactive protein; FC, fecal calprotectin; endoscopic activity was assessed using SES-CD in Crohn’s disease (remission 0–2, active ≥ 3, moderate and severe ≥ 7) and mMES in ulcerative colitis (remission 0–2, active ≥ 3, moderate and severe ≥ 7); asterisks (*) denote significant *p*-values (*p* < 0.05).

**Table 4:** Multivariate analysis of combination of biomarkers by logistic regression.

	AUC (95% CI) (Sens;Spec)	Dg. Accuracy, %
CD (remission vs. active)		
C4M, C3M/PRO-C3	0.67(0.40–0.81) (54;73)	64.00
C4M, C3M/PRO-C3 ^a^	0.80 (0.66–0.90) (75;84)	75.51
CD (remission vs. moderate to severe)		
C4M, C3M/PRO-C3	0.77 (0.60–0.89) (57;85)	75.00
C4M, C3M/PRO-C3 ^a^	0.93 (0.66–0.90) (93;80)	84.62
UC (remission vs. active)		
C3M, C4M	0.66 (0.43–0.85) (88;60)	76.19
C3M, C4M ^a^	0.95 (0.79–1.00) (94;80)	90.48
C4M, C3M/PRO-C3	0.65 (0.45–0.86) (44;70)	76.19
C4M, C3M/PRO-C3 ^a^	0.93 (0.72–0.99) (88;100)	85.71
UC (remission vs. moderate to severe)		
C3M, C4M	0.80 (0.48–0.96) (43;80)	75.00
C4M, C3M/PRO-C3	0.94 (0.65–0.99) (86;100)	83.33

Endoscopic activity was assessed using SES-CD in Crohn’s disease (CD) (remission 0–2, active ≥ 3, moderate to severe ≥ 7), and mMES in ulcerative colitis (UC) (remission 0–2, active ≥ 3, moderate to severe ≥ 7); ^a^ regression model was adjusted for confounding factors: in CD for age, gender, body mass index (BMI), smoking, prior surgery, biological therapy, Montreal classification, and in UC for age, gender, BMI, biological therapy, Montreal classification; on discriminating moderate to severe disease and remission in UC, no adjustments were made due to small sample size.

## Data Availability

The data presented in this study are available on reasonable request from the corresponding author. The data are not publicly available due to ethical considerations.

## Supplementary Materials

The following supporting information can be downloaded at: https://www.mdpi.com/article/10.3390/jcm11195907/s1, Figure S1: Comparison of ECM biomarker levels according to endoscopic disease activity (SES-CD) in Crohn’s disease; Figure S2: Comparison of ECM biomarker levels according to endoscopic disease activity (mMES) in ulcerative colitis patients; Figure S3: Depiction of type III, IV. and V collagen remodeling in Crohn’s disease and differences between healthy donors, remission, and active disease; Figure S4: Depiction of type III, IV, and V collagen remodeling in ulcerative colitis, and differences between healthy donors, remission, and active disease; Figure S5: Principal component analysis of collagen biomarkers.

## References

[1] Gomollón F., Dignass A., Annese V., Tilg H., Van Assche G., Lindsay J.O., Peyrin-Biroulet L., Cullen G.J., Daperno M., Kucharzik T. (2017). 3rd European evidence-based consensus on the diagnosis and management of Crohn’s disease 2016. Part 1: Diagnosis and medical management. J. Crohns Colitis.

[2] Magro F., Gionchetti P., Eliakim R., Ardizzone S., Armuzzi A., Barreiro-de Acosta M., Burisch J., Gecse K.B., Hart A.L., Hindryckx P. (2017). Third European evidence-based consensus on diagnosis and management of ulcerative colitis. Part 1: Definitions, diagnosis, extra-intestinal manifestations, pregnancy, cancer surveillance, surgery, and ileo-anal pouch disorders. J. Crohns Colitis.

[3] Iborra M., Beltrán B., Nos P. (2016). Noninvasive testing for mucosal inflammation in inflammatory bowel disease. Gastrointest. Endosc. Clin. N. Am..

[4] Mosli M., Fahmy M., Garg S.K., Feagan S.G., Baker K.A., Zou G.Y., MacDonald J.K., Sandborn W.J., Chande N. (2013). Biomarkers for assessing disease activity in inflammatory bowel disease. Cochrane Database Syst Rev..

[5] Mao R., Xiao Y.L., Gao X., Chen B.L., He Y., Yang L., Hu P.J., Chen M.H. (2012). Fecal calprotectin in predicting relapse of inflammatory bowel diseases: A meta-analysis of prospective studies. Inflamm. Bowel Dis..

[6] Costa F., Mumolo M.G., Ceccarelli L., Bellini M., Romano M.R., Sterpi C., Ricchiuti A., Marchi S., Bottai M. (2005). Calprotectin is a stronger predictive marker of relapse in ulcerative colitis than in Crohn’s disease. Gut.

[7] Louis E. (2015). Fecal calprotectin: Towards a standardized use for inflammatory bowel disease management in routine practice. J. Crohns Colitis.

[8] Van Rheenen P.F., Van de Vijver E., Fidler V. (2010). Faecal calprotectin for screening of patients with suspected inflammatory bowel disease: Diagnostic meta-analysis. BMJ.

[9] D’Haens G., Ferrante M., Vermeire S., Baert F., Noman M., Moortgat L., Geens P., Iwens D., Aerden I., Van Assche G. (2012). Fecal calprotectin is a surrogate marker for endoscopic lesions in inflammatory bowel disease. Inflamm. Bowel Dis..

[10] Goutorbe F., Goutte M., Minet-Quinard R., Boucher A.L., Pereira B., Bommelaer G., Buisson A. (2015). Endoscopic factors influencing fecal calprotectin value in Crohn’s disease. J. Crohns Colitis.

[11] Dumoulin E.N., Van Biervliet S., Langlois M.R., Delanghe J.R. (2015). Proteolysis is a confounding factor in the interpretation of faecal calprotectin. Clin. Chem. Lab. Med..

[12] Du L., Foshaug R., Huang V.W., Kroeker K.I., Dieleman L.A., Halloran B.P., Wong K., Fedorak R.N. (2018). Within-stool and within-day sample variability of fecal calprotectin in patients with inflammatory bowel disease. J. Clin. Gastroenterol..

[13] Meling T., Aabakken L., Roseth A., Osnes M. (1996). Faecal calprotectin shedding after short-term treatment with non-steroidal anti-inflammatory drugs. Scand. J. Gastroenterol..

[14] Mumolo M.G., Bertani L., Ceccarelli L., Laino G., Di Fluri G., Albano E., Tapete G., Costa F. (2018). From bench to bedside: Fecal calprotectin in inflammatory bowel diseases clinical setting. World J. Gastroenterol..

[15] Bonnans C., Chou J., Werb Z. (2014). Remodelling the extracellular matrix in development and disease. Nat. Rev. Mol. Cell. Biol..

[16] Yurchenco P.D., Schittny J.C. (1990). Molecular architecture of basement membranes. FASEB J..

[17] Bosman F.T., Stamenkovic I. (2003). Functional structure and composition of the extracellular matrix. J. Pathol..

[18] Orlichenko L.S., Radisky D.C. (2008). Matrix metalloproteinases stimulate epithelial-mesenchymal transition during tumor development. Clin. Exp. Metastasis.

[19] Baugh M.D., Perry M.J., Hollander A.P., Davies D.R., Cross S.S., Lobo A.J., Taylor C.J. (1999). Evans GS Matrix metalloproteinase levels are elevated in inflammatory bowel disease. Gastroenterology.

[20] Mortensen J.H., Godskesen L.E., Jensen M.D., Van Haaften W.T., Klinge L.G., Olinga P., Dijkstra G., Kjeldsen J., Karsdal M.A., Bay-Jensen A.C. (2015). Fragments of citrullinated and MMP-degraded vimentin and MMP-degraded type III collagen are novel serological biomarkers to differentiate Crohn’s disease from ulcerative colitis. J. Crohns Colitis.

[21] Van Haaften W.T., Mortensen J.H., Karsdal M.A., Bay-Jensen A.C., Dijkstra G., Olinga P. (2017). Misbalance in type III collagen formation/degradation as a novel serological biomarker for penetrating (Montreal B3) Crohn’s disease. Aliment. Pharmacol. Ther..

[22] Goffin L., Fagagnini S., Vicari A., Mamie C., Melhem H., Weder B., Lutz C., Lang S., Scharl M., Rogler G. (2016). Anti-MMP-9 antibody: A promising therapeutic strategy for treatment of inflammatory bowel disease complications with fibrosis. Inflamm. Bowel Dis..

[23] Mortensen J.H., Manon-Jensen T., Jensen M.D., Hägglund P., Klinge L.G., Kjeldsen J., Krag A., Karsdal M.A., Bay-Jensen A.C. (2017). Ulcerative colitis, Crohn’s disease, and irritable bowel syndrome have different profiles of extracellular matrix turnover, which also reflects disease activity in Crohn’s disease. PLoS ONE.

[24] Graham M.F., Diegelmann R.F., Elson C.O., Lindblad W.J., Gotschalk N., Gay S., Gay R. (1988). Collagen content and types in the intestinal strictures of Crohn’s disease. Gastroenterology.

[25] Naito Y., Yoshikawa T. (2005). Role of matrix metalloproteinases in inflammatory bowel disease. Mol. Aspects Med..

[26] Mortensen J.H., Lindholm M., Langholm L.L., Kjeldsen J., Bay-Jensen A.C., Karsdal M.A., Manon-Jensen T. (2019). The intestinal tissue homeostasis—The role of extracellular matrix remodeling in inflammatory bowel disease. Expert Rev. Gastroenterol. Hepatol..

[27] Karsdal M.A., Nielsen S.H., Leeming D.J., Langholm L.L., Nielsen M.J., Manon-Jensen T., Siebuhr A., Gudmann N.S., Rønnow S., Sand J.M. (2017). The good and the bad collagens of fibrosis—The role in signaling and organ function. Adv. Drug Deliv. Rev..

[28] Zhen E.Y., Brittain I.J., Laska D.A., Mitchell P.G., Sumer E.U., Karsdal M.A., Duffin K.L. (2008). Characterization of metalloprotease cleavage products of human articular cartilage. Arthritis Rheum..

[29] Karsdal M.A., Henriksen K., Leeming D.J., Mitchell P., Duffin K., Barascuk N., Klickstein L., Aggarwal P., Nemirovskiy O., Byrjalsen I. (2009). Biochemical markers and the FDA Critical Path: How biomarkers may contribute to the understanding of pathophysiology and provide unique and necessary tools for drug development. Biomarkers.

[30] Karsdal M.A., Henriksen K., Leeming D.J., Woodworth T., Vassiliadis E., Bay-Jensen A.C. (2010). Novel combinations of Post-Translational Modification (PTM) neo-epitopes provide tissue-specific biochemical markers—Are they the cause or the consequence of the disease?. Clin. Biochem..

[31] Karsdal M.A., Nielsen M.J., Sand J.M., Henriksen K., Genovese F., Bay-Jensen A.C., Smith V., Adamkewicz J.I., Christiansen C., Leeming D.J. (2013). Extracellular Matrix Remodeling: The common denominator in connective tissue diseases. Possibilities for evaluation and current understanding of the matrix as more than a passive architecture, but a key player in tissue failure. Assay Drug Dev. Technol..

[32] Barascuk N., Veidal S.S., Larsen L., Larsen D.V., Larsen M.R., Wang J., Zheng Q., Xing R., Cao Y., Rasmussen L.M. (2010). A novel assay for extracellular matrix remodeling associated with liver fibrosis: An enzyme-linked immunosorbent assay (ELISA) for a MMP-9 proteolytically revealed neo-epitope of type III collagen. Clin. Biochem..

[33] Sand J.M., Larsen L., Hogaboam C., Martinez F., Han M., Røssel Larsen M., Nawrocki A., Zheng Q., Karsdal M.A., Leeming D.J. (2013). MMP mediated degradation of type IV collagen alpha 1 and alpha 3 chains reflects basement membrane remodeling in experimental and clinical fibrosis—Validation of two novel biomarker assays. PLoS ONE.

[34] Nielsen M.J., Nedergaard A.F., Sun S., Veidal S.S., Larsen L., Zheng Q., Suetta C., Henriksen K., Christiansen C., Karsdal M.A. (2013). The neo-epitope specific PRO-C3 ELISA measures true formation of type III collagen associated with liver and muscle parameters. Am. J. Transl. Res..

[35] Leeming D.J., Nielsen M.J., Dai Y., Veidal S.S., Vassiliadis E., Zhang C., He Y., Vainer B., Zheng Q., Karsdal M.A. (2012). Enzyme-linked immunosorbent serum assay specific for the 7S domain of collagen type IV (P4NP 7S): A marker related to the extracellular matrix remodeling during liver fibrogenesis. Hepatol. Res..

[36] Vassiliadis E., Veidal S.S., Simonsen H., Larsen D.V., Vainer B., Chen X., Zheng Q., Karsdal M.A., Leeming D.J. (2011). Immunological detection of the type V collagen propeptide fragment, PVCP-1230, in connective tissue remodeling associated with liver fibrosis. Biomarkers.

[37] Lobatón T., Bessissow T., De Hertogh G., Lemmens B., Maedler C., Van Assche G., Vermeire S., Bisschops R., Rutgeerts P., Bitton A. (2015). The Modified Mayo Endoscopic Score (MMES): A new index for the assessment of extension and severity of endoscopic activity in ulcerative colitis patients. J. Crohns Colitis.

[38] Karsdal M.A., Hjuler S.T., Luo Y., Rasmussen D.G.K., Nielsen M.J., Holm Nielsen S., Leeming D.J., Goodman Z., Arch R.H., Patel K. (2019). Assessment of liver fibrosis progression and regression by a serological collagen turnover profile. Am. J. Physiol. Gastrointest. Liver Physiol..

[39] Lindholm M., Manon-Jensen T., Madsen G.I., Krag A., Karsdal M.A., Kjeldsen J., Mortensen J.H. (2019). Extracellular matrix fragments of the basement membrane and the interstitial matrix are serological markers of intestinal tissue remodeling and disease activity in dextran sulfate sodium colitis. Dig. Dis. Sci..

[40] Van Haaften W.T., Mortensen J.H., Dige A.K., Grønbæk H., Hvas C.L., Bay-Jensen A.C., Karsdal M.A., Olinga P., Manon-Jensen T., Dijkstra G. (2020). Serological biomarkers of tissue turnover identify responders to anti-TNF therapy in Crohn’s disease: A pilot study. Clin. Transl. Gastroenterol..

