# Supplementary material for: Inflammatory Biomarkers of Extracellular Matrix Remodeling and Disease Activity in Crohn’s Disease and Ulcerative Colitis
Source: J Clin Med. 2022 Oct 7;11(19):5907. doi: 10.3390/jcm11195907 (PMC9572110; doi:10.3390/jcm11195907)
Supplement: Supplementary file 1 [file jcm-11-05907-s001.zip › jcm-1905059-supplementary.pdf]

SES-CD

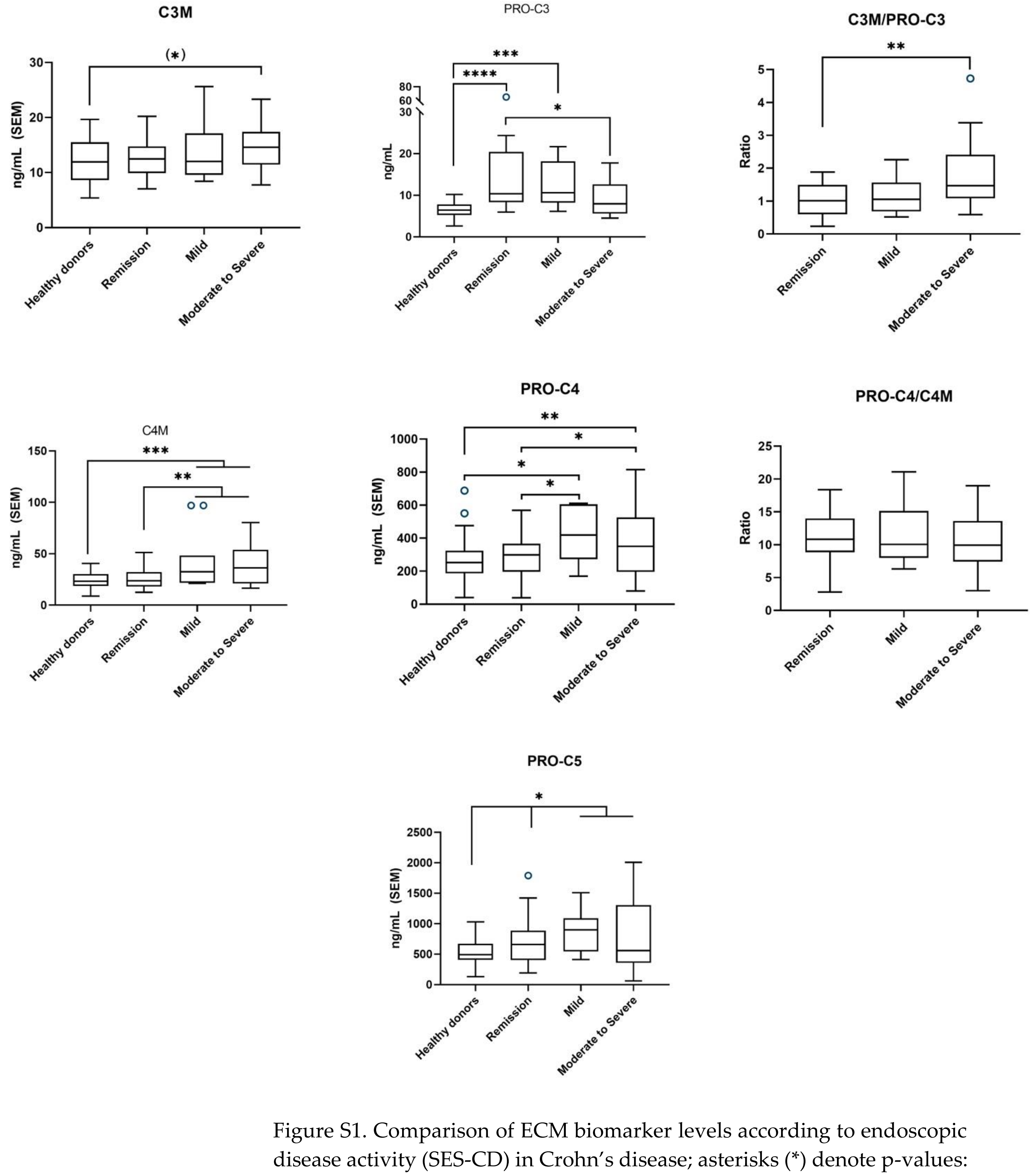

Figure S1. Comparison of ECM biomarker levels according to endoscopic disease activity (SES-CD) in Crohn’s disease; asterisks (*) denote p-values: *p<0.05, **p<0.01, ***p<0.001, (*)p<0.1; endoscopic activity was assessed using SES-CD (remission 0-2, active ≥3, moderate to severe ≥7). N of patients (remission 32; mild 10; moderate to severe 18; healthy donors 29)

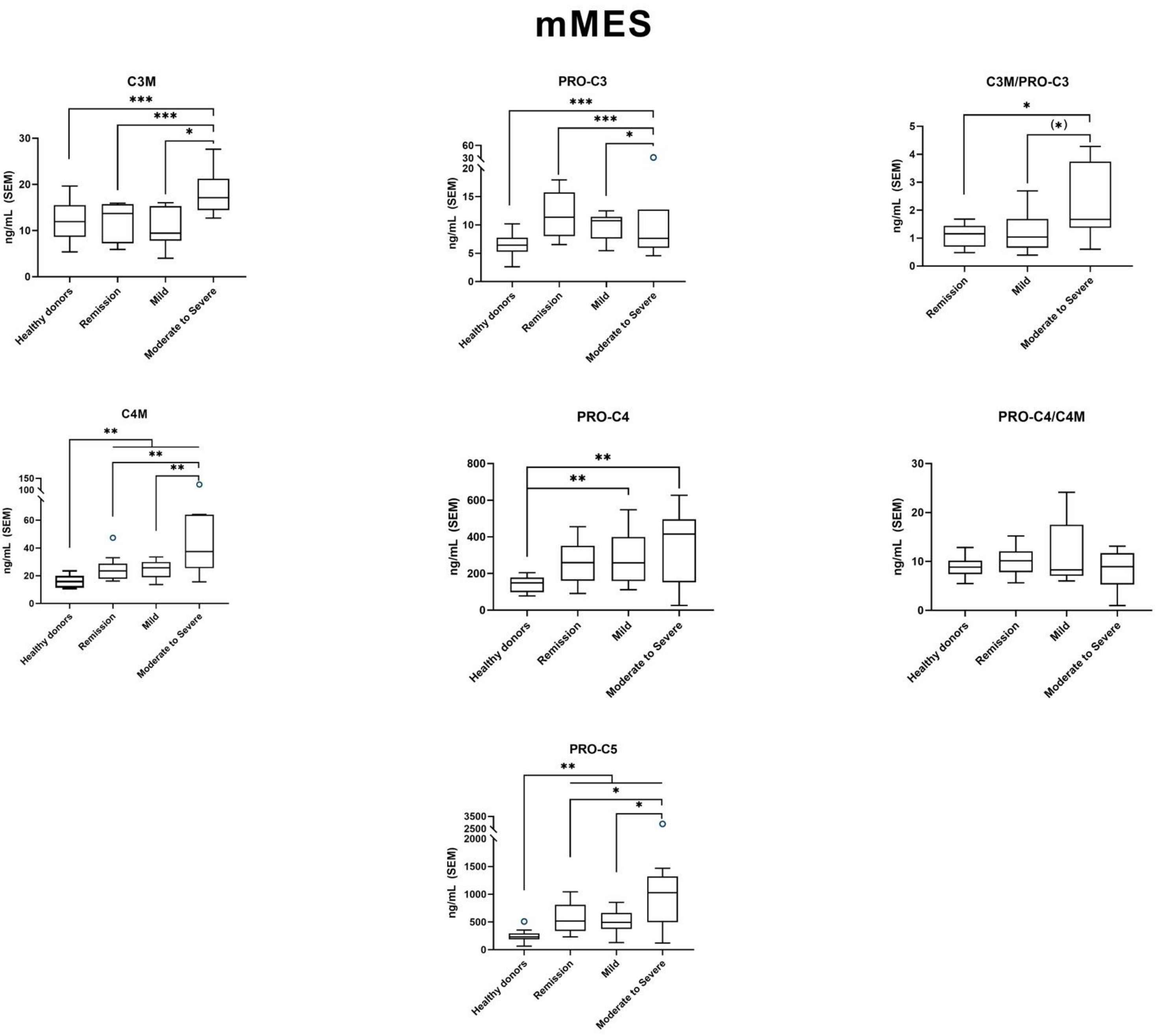

Figure S2. Comparison of ECM biomarker levels according to endoscopic disease activity (mMES) in ulcerative colitis patients; asterisks (*) denote p-values: *p<0.05, **p<0.01, ***p<0.001, (*)p<0.1; endoscopic activity was assessed using mMES (remission 0-2, active ≥3, moderate to severe ≥7). N of patients (remission 10; mild 13; moderate to severe 10; healthy donors 29)

CD: Clinical and Biochemical disease activity

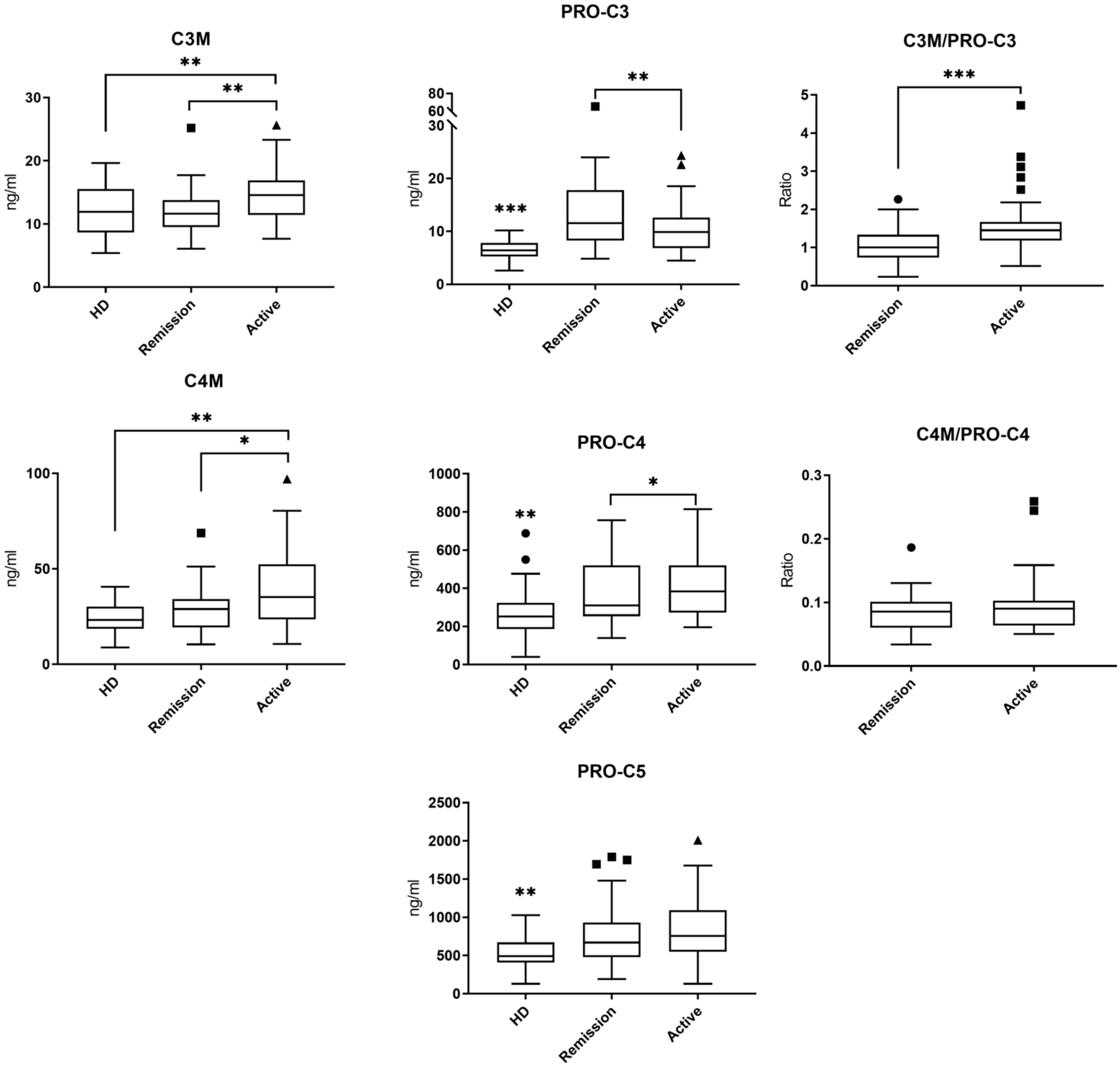

Figure S3. Depiction of type III, IV and V collagen remodeling in Crohn’s disease and differences between healthy donors, remission and active disease; asterisks (*) denote p-values: *p<0.05, **p<0.01, ***p<0.001; clinical and biochemical activity was defined as CDAI ≥150 or C-reactive protein (CRP) >5 mg/L, N of patients (remission 51; active 59; healthy donors 29)

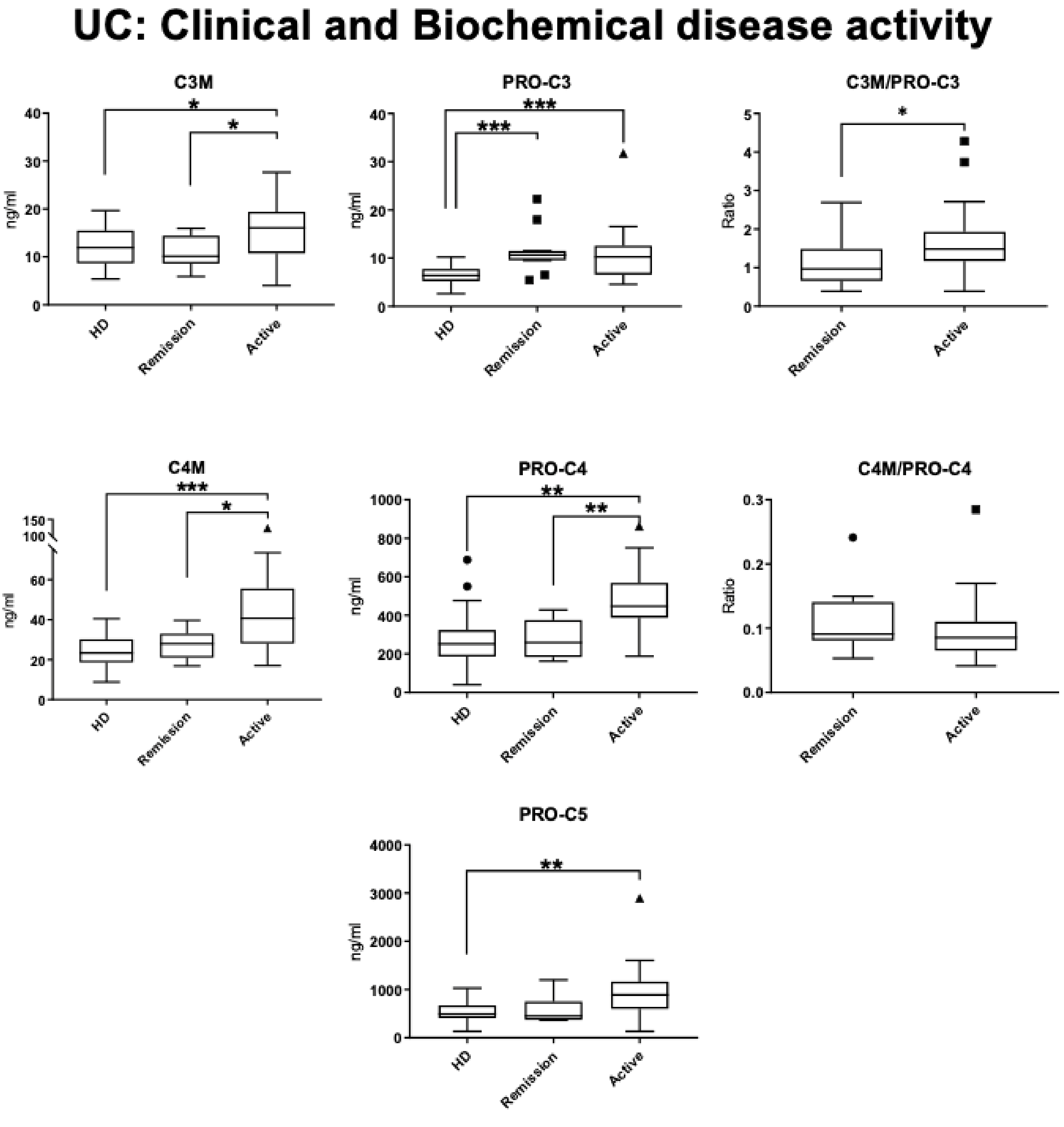

Figure S4. Depiction of type III, IV and V collagen remodeling in ulcerative colitis, and differences between healthy donors, remission and active disease; asterisks (*) denote p-values: *p<0.05, **p<0.01, ***p<0.001; clinical and biochemical activity was defined as pMayo >1 or CRP >5 mg/L. N of patients (remission 22; active 30; healthy donors 29

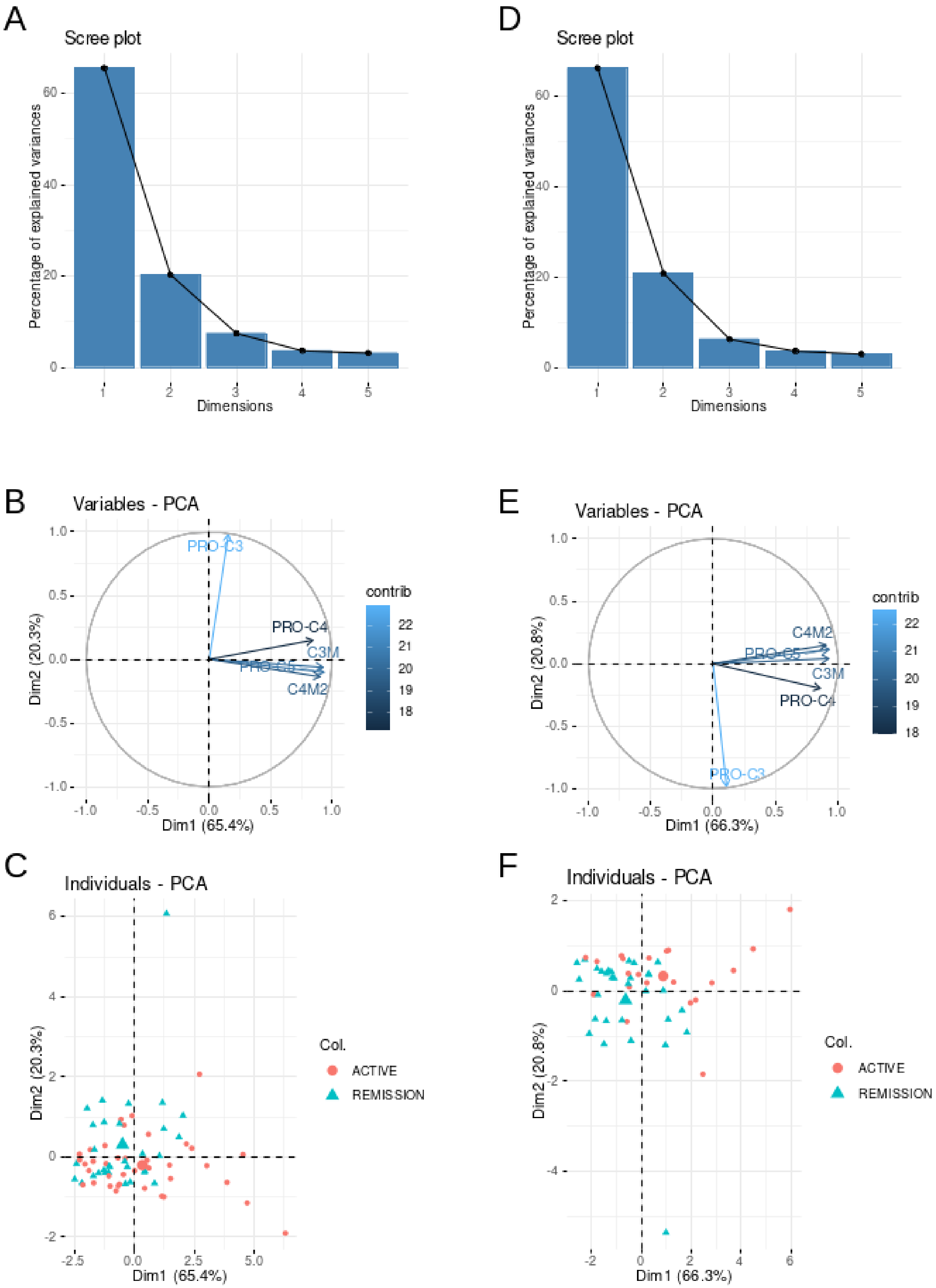
A
Scree plot
Percentage of explained variances
Dimensions
B
Variables - PCA
PRO-C3
PRO-C4
C3M
PRO-C5
C4M2
contrib
Dim1 (65.4%)
Dim2 (20.3%)
C
Individuals - PCA
Col.
ACTIVE
REMISSION
Dim1 (65.4%)
Dim2 (20.3%)
D
Scree plot
Percentage of explained variances
Dimensions
E
Variables - PCA
C4M2
PRO-C5
C3M
PRO-C4
PRO-C3
contrib
Dim1 (66.3%)
Dim2 (20.8%)
F
Individuals - PCA
Col.
ACTIVE
REMISSION
Dim1 (66.3%)
Dim2 (20.8%)

Figure S5. Principal component analysis (PCA) of collagen biomarkers. A, B, C – PCA analysis of remission vs. active endoscopic disease; D, E, F – PCA analysis of remission vs. moderate to severe endoscopic disease
